# Paraptosis and NF-κB activation are associated with protopanaxadiol-induced cancer chemoprevention

**DOI:** 10.1186/1472-6882-13-2

**Published:** 2013-01-03

**Authors:** Chong-Zhi Wang, Binghui Li, Xiao-Dong Wen, Zhiyu Zhang, Chunhao Yu, Tyler D Calway, Tong-Chuan He, Wei Du, Chun-Su Yuan

**Affiliations:** 1 Tang Center for Herbal Medicine Research, University of Chicago, Chicago, IL, USA; 2 Department of Anesthesia and Critical Care, University of Chicago, Chicago, IL, USA; 3 Ben May Department for Cancer Research, University of Chicago, Chicago, IL, USA; 4 Department of Surgery, University of Chicago, Chicago, IL, USA; 5 Committee on Clinical Pharmacology and Pharmacogenomics, University of Chicago, Chicago, IL, USA; 6 Tang Center for Herbal Medicine Research, and Department of Anesthesia & Critical Care, Pritzker School of Medicine, University of Chicago, 5841 S. Maryland Ave., MC 4028, Chicago, IL, 60637, USA

**Keywords:** Ginseng, Protopanaxadiol, PPD, Paraptosis, Cytoplasmic vacuoles, Mitochondrial swelling, Antioxidant, Cancer chemoprevention

## Abstract

**Background:**

Protopanaxadiol (PPD) is a triterpenoid that can be prepared from steamed ginseng. PPD possesses anticancer potential via caspase-dependent apoptosis. Whether paraptosis, a type of the caspase-independent cell death, is also induced by PPD has not been evaluated.

**Methods:**

Cell death, the cell cycle and intracellular reactive oxygen species (ROS) were analyzed by flow cytometry after staining with annexin V/PI, PI/RNase or H2DCFDA. We observed morphological changes by crystal violet staining assay. Mitochondrial swelling was measured by ultraviolet–visible spectrophotometry. The activation of NF-κB was measured by luciferase reporter assay.

**Results:**

At comparable concentrations of 5-fluorouracil, PPD induced more cell death in human colorectal cancer cell lines HCT-116 and SW-480. We demonstrated that PPD induced paraptosis in these cancer cells. PPD treatment significantly increased the percentage of cancer cells with cytoplasmic vacuoles. After the cells were treated with PPD and cycloheximides, cytoplasmic vacuole generation was inhibited. The paraptotic induction effect of PPD was also supported by the results of the mitochondrial swelling assay. PPD induced ROS production in cancer cells, which activated the NF-κB pathway. Blockage of ROS by NAC or PS-1145 inhibited the activation of NF-κB signaling.

**Conclusions:**

PPD induces colorectal cancer cell death in part by induction of paraptosis. The anticancer activity of PPD may be enhanced by antioxidants such as green tea, which also inhibit the activation of NF-κB signaling.

## Background

The clinical management of cancer invariably involves diverse conventional modalities, including surgery, radiation, and chemotherapy [1]. Because of the complexity of human cancer, alternative management may be needed to improve the efficacy of therapeutic treatments and the quality of life of patients [2]. Cancer chemoprevention or treatment may combine natural products with chemotherapeutic agents to inhibit tumor development [3-5]. Natural products have been shown to be one option for cancer chemoprevention and new drug development [6-8].

Long-term consumption of certain botanicals, such as ginseng, is associated with a reduction in cancer incidence in humans [9,10]. Anticancer potential has been observed with ginseng and its compounds, including the enhancement of 5-fluoruracil’s anti-proliferative effects on human cancer cells [11-13]. Steaming ginseng changes its ginsenoside profile and increases its anticancer potential [14,15].

**Figure 1:**
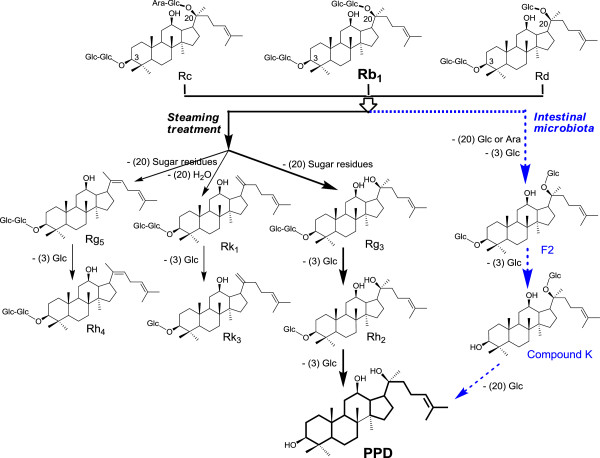
**Transformation pathways of panaxadiol group ginsenosides Rb1, Rc and Rd by steaming treatment and intestinal microbiota.** During steaming, ginsenosides Rb1, Rc and Rd are mainly transformed to Rg5, Rk1, and Rg3. Rg3 then converts to Rh2 and PPD. In addition, intestinal microbiota metabolize Rb1, Rc and Rd to compound K, which can be further converted to PPD (dotted lines).

The ginsenoside Rh2 in ginseng induced apoptosis and paraptosis-like cell death in colon cancer cells [16]. Ginsenoside Rh2 levels are low in untreated ginseng, but after steaming, Rh2 levels increase [17]. In a recent review of the relationship between the structure and function of ginsenosides, we proposed that reducing sugar molecules in ginsenosides increases their anticancer bioactivity [7]. We also observed that further heat treatment of ginseng initiated the conversion of Rh2 to protopanaxadiol (PPD). In this transformation, another sugar molecule is removed from Rh2 (see Discussion and Figure 1).

PPD possesses anticancer potential because it induces cell apoptosis, a programmed cell death that is caspase-dependent [18]. Paraptosis, another type of cell death, is characterized by the accumulation of cytoplasmic vacuoles and mitochondrial swelling [19]. Whether PPD-induced cell death also is mediated by caspase-independent paraptosis, like Rh2, is not known. In previous studies, Rh2 increased levels of reactive oxygen species (ROS) and activated the NF-κB survival pathway [16]. It would be interesting to know whether ROS blockage and inhibition of NF-κB signaling increases PPD-induced cell death and whether PPD’s effect is enhanced by antioxidants because antioxidant dietary supplements are often self-administered by cancer patients [20,21]. The present study data suggest that paraptosis and NF-κB activation are associated with PPD-induced cancer chemoprevention.

## Methods

### Chemicals and reagents

DMSO and other solvents were obtained from Fisher Scientific (Pittsburgh, PA). Trypsin, McCoy’s 5A, Leibovitz’s L-15 medium, fetal bovine serum (FBS), and penicillin/streptomycin solution (200×) were obtained from Mediatech, Inc. (Herndon, VA). N-Acetyl-L-cysteine (NAC), PS-1145, propidium iodide (PI) and RNase were obtained from Sigma (St. Louis, MO). NAC, which is an antioxidant, was dissolved in the growth medium. PS-1145, a specific inhibitor of the NF-κB pathway, was dissolved in DMSO as a 20 mM stock buffer. Protopanaxadiol (PPD) was obtained from National Institutes for Food and Drug Control (Beijing, China). 5-Fluorouracil (5-FU) was obtained from American Pharmaceutical Partners Inc. (Schaumburg, IL). Luciferase assay kits were obtained from Promega (Madison, WI). Annexin V Apoptosis Kit was purchased from BD Biosciences (San Diego, CA). Reactive oxygen species (ROS) dye 2^′^,7^′^-dichlorodihydrofluorescein diacetate (H2DCFDA) and L-glutamine were obtained from Invitrogen (Carlsbad, CA).

### Cell culture

Human colorectal cancer cells HCT-116 and SW-480 were obtained from the American Type Culture Collection (ATCC, Manassas, VA), and were maintained in McCoy’s 5A (HCT-116) or Leibovitz’s L-15 (SW-480) medium supplemented with 5% fetal bovine serum, 50 IU of penicillin/streptomycin and 2 mmol/L of L-glutamine in a humidified atmosphere with 5% CO_2_ at 37°C.

### Cell death assay

Cells were seeded into 24-well plates (2 × 10^5^ cells/well). Samples were prepared based on the instruction provided with the Annexin V Apoptosis Kit. Briefly, after treatment as indicated in the result section, the adherent and detached cells were collected and washed twice with binding buffer containing 10 mM HEPES, pH 7.4, 140 mM NaCl, 2.5 mM CaCl, and then 1 × 10^5^ cells were resuspended in 100 μl of binding buffer. 5 μl of annexin V-FITC and 10 μl of propidium iodide (50 μg/ml, stocking concentration) were added to the cell suspension. After gently mixing, the cells were incubated for 15 min at room temperature, and then 400 μl of binding buffer was added to get the sample ready. Quantification of cell death was performed using a FACScan flow cytometer (BD Biosciences, San Jose, CA). All the data analyses were performed using FlowJo analysis software, version 6.0 (FlowJo LLC, Ashland, OR). Annexin V-positive and/or PI-positive cells were considered as cell death.

### Cell cycle analysis

Cells were seeded in 24-well plates (2 × 10^5^ cells/well). After 48 h of drug exposure or control conditions, cells were fixed gently by adding 80% ethanol before they were placed in a freezer for 2 h; then cells were treated with 0.25% Triton X-100 for 5 min in an ice bath. The cells were resuspended in 130 μl of PBS. Then, 5 μl of PI/RNase staining solution was added to the cell suspension. Cells were incubated in a dark room for 10 min at room temperature and analyzed using a FACScan flow cytometer.

### Crystal violet staining assay

HCT-116 cells were seeded in 24-well plates (1 × 10^5^ cells/well). After 12 h of drug exposure or control conditions, the medium was removed and the cells were washed and stained with 0.2% crystal violet in 10% phosphate-buffered formaldehyde for 2 min. The staining solution was removed and the cells were washed twice with PBS. The remaining cells adhering to the wells were observed under the microscope and photographed.

### Mitochondrial swelling assay

Human liver mitochondria were obtained from Xenotech LLC (Lenexa, KS). Before the experiment, the mitochondria were suspended in 230 mM mannitol, 70 mM sucrose, 10 mM Tris–HCl, and 1 mM EDTA, with a protein concentration of 0.5 mg/ml. After exposure to 35 μM of PPD or control condition at 25°C, mitochondrial swelling was measured by the ultraviolet–visible spectrophotometry method. The absorbance changes at 540 nm were monitored [22].

### Intracellular ROS assay

Intracellular ROS production was monitored by the permeable fluorescence dye, H2DCFDA. H2DCFDA can readily react with ROS to form the fluorescent product 2,7-dichlorofluorescein (DCF). The intracellular fluorescence intensity of DCF is proportional to the amount of ROS generated by the cells. After the indicated treatment, the cells were incubated with 10 μM of H2DCFDA for thirty minutes and then cells were harvested and resuspended in PBS (10^6^ cells/mL). The fluorescence intensity of intracellular DCF (excitation 488 nm, emission 530 nm) was measured using a FACScan flow cytometer.

### Luciferase activity assay

The plasmids containing the luciferase reporter gene with or without a NF-κB response element and phRL-TK plasmid for the transfection control were donated by Liao’s lab (Ben May Department for Cancer Research, University of Chicago). 10^4^ cells were seeded into 48-well plates for 24 h and were co-transfected with 0.5 μg of plasmid containing report construct and 10 ng of phRL-TK using transfection reagent Effectene (Qiagen, Chatsworth, CA). At 24 h post-transfection, the cells were treated as desired. Luciferase activity was measured with a commercial kit (Promega Dual luciferase II) on a Monolight luminometer (Becton Dickinson, Franklin Lakes, NJ).

### Statistical analysis

All experiments were performed in triplicate. The data are presented as mean ± standard error (SE). A one-way ANOVA determined whether the results had statistical significance. In some cases, a Student’s *t*-test was used for comparing two groups. The level of statistical significance was set at *P* < 0.05.

## Results

### Effects of PPD on cell death and cell cycle of human colorectal cancer cells

**Figure 2:**
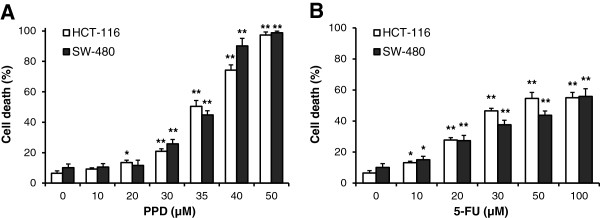
**Effects of PPD and 5-FU on cell death in two human colorectal cancer cell lines.** (**A**) Effects of PPD (10–50 μM) on HCT-116 and SW-480 cell death. (**B**) Effects of 5-FU (10–100 μM) on HCT-116 and SW-480 cell death. Cell death in the presence of PPD or 5-FU was measured after 48 h treatment. Data are presented as the mean ± standard error. *P < 0.05, **P < 0.01 vs. control.

We used two human colorectal cancer cell lines, HCT-116 and SW-480, to evaluate the effect of PPD on induction of cell death. As shown in Figure 2, for HCT-116 cells, treatment with 30, 35 and 40 μM of PPD for 48 h induced 20.9 ± 1.7%, 50.4 ± 3.9% and 74.2 ± 3.4% cell death, respectively (all *P* < 0.01 vs. control, 6.5 ± 1.5%). Similar effects were observed in SW-480 cells (Figure 2A). PPD significantly induced cell death in both of the cell lines in a concentration-dependent manner. As a positive control, we also tested the effect of 5-FU, a commonly used chemotherapeutic agent against colorectal cancer, on the induction of cell death (Figure 2B). The data show that, when comparable concentrations were used, the effect of PPD was stronger than that of 5-FU, suggesting that PPD could be a potential colorectal cancer chemopreventive compound.

**Figure 3:**
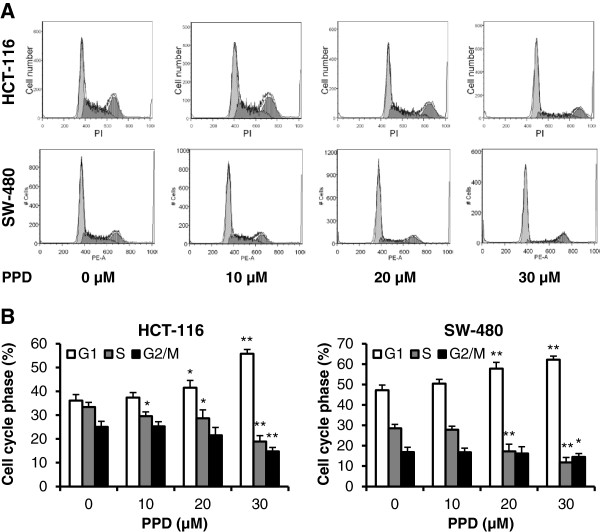
**Effects of PPD on colorectal cancer cell cycle.** HCT-116 and SW-480 cells were treated with 10–30 μM PPD for 48 h, then fixed in ethanol and stained with propidium iodide. DNA content was determined by flow cytometry. (**A**) Representative histograms of DNA content in each experimental group. (**B**) Percentage of each cell cycle phase with various treatments. Data are presented as the mean ± standard error. *P < 0.05, **P < 0.01 vs. control.

PPD’s effect on the cancer cell cycle is illustrated in Figure 3. Compared to control (G1 36.1%, S 33.4% and G2/M 25.1% for HCT-116; G1 47.2%, S 28.5% and G2/M 16.9% for SW-480), 10 and 20 μM of PPD did not change the cell cycle profile. Treatment with 30 μM of PPD for 48 h changed the cell cycle profile, with G1 55.8%, S 18.9% and G2/M 14.8% for HCT-116 cells; G1 62.2%, S 11.8% and G2/M 14.5% for SW-480. In both HCT-116 and SW-480 cells, PPD obviously increased cell fractions in the G1 phase.

### PPD-induced paraptosis-like cell death

**Figure 4:**
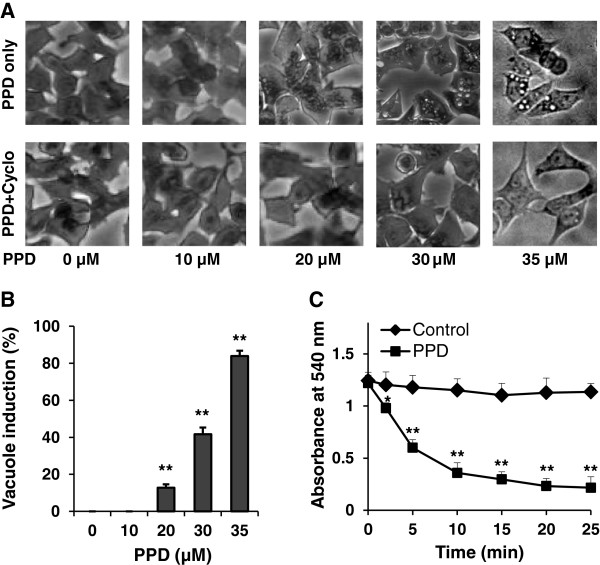
**Induction of PPD on paraptosis-like cell death.** (**A**) HCT-116 cells were treated with 10–35 μM of PPD in combination with or without 10 μg/ml of cycloheximide (Cyclo) for 24 h, and then pictured. (**B**) The percentage of cytoplasmic vacuoles with 10–35 μM of PPD. (**C**) Mitochondrial swelling induced by 35 μM of PPD observed by monitoring absorbance at 540 nm in isolated human liver mitochondria. Data are presented as the mean ± standard error. *P < 0.05, **P < 0.01 vs. control.

To explore the potential mechanism through which PPD induced cell death, morphological observation was conducted after crystal violet staining. As shown in Figure 4A, after treatment with 35 μM of PPD for 24 h, cytoplasmic vacuolization was significant. HCT-116 cells treated with vehicle control did not show vacuoles. When the PPD concentration was increased, the fraction of cells with cytoplasmic vacuolization increased (Figure 4B). Visible cytoplasmic vacuolization is a typical feature of paraptosis. Induction of paraptosis requires new protein synthesis but resists inhibition by caspase inhibitors. Pre-treatment of HCT-116 cells with cycloheximide (Cyclo), a protein synthesis inhibitor, blocked PPD-induced vacuole formation (Figure 4A). In addition to the formation of vacuoles in the cytoplasm, paraptosis is also characterized by mitochondrial swelling [8]. As illustrated in Figure 4C, PPD significantly induced mitochondrial swelling by monitoring absorbance at 540 nm in isolated human liver mitochondria. Thus, PPD induced cancer cell death in part via paraptosis.

### Effects of PPD on induction of ROS

**Figure 5:**
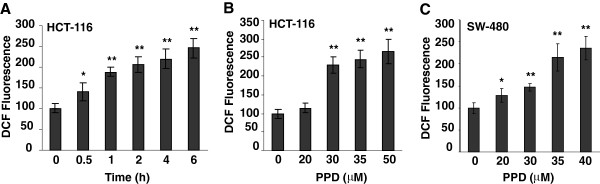
**Effects of PPD on the induction of ROS on colorectal cancer cells.** (**A**) HCT-116 cells were treated with 35 μM of PPD, and then ROS level was measured at different time points. (**B**) HCT-116 cells were treated with different concentrations of PPD for 6 h, and then ROS level was determined. (**C**) SW-480 cells were treated with different concentrations of PPD for 6 h, and then ROS level was determined. Data are presented as the mean ± standard error. *P < 0.05, **P < 0.01 vs. control.

We measured ROS levels in colorectal cancer cells after treatment with PPD to determine if the compound induced ROS. To evaluate time-dependent activity, HCT-116 cells were treated with 35 μM of PPD, and ROS levels were measured at different time points. After treatment with PPD for 6 h, ROS level was significantly higher than in the control. Thus, the 6 h time point was chosen (Figure 5A). Using two cell lines, HCT-116 and SW-480, ROS levels were determined after treatment with 20–40 μM of PPD. As shown in Figure 5B and 5C, PPD significantly induced ROS production at 6 h in a concentration-dependent manner in both cell lines. These results suggest that PPD induced ROS production in human colorectal cancer cells.

### PPD activated the NF-κB pathway linked to ROS generation

**Figure 6:**
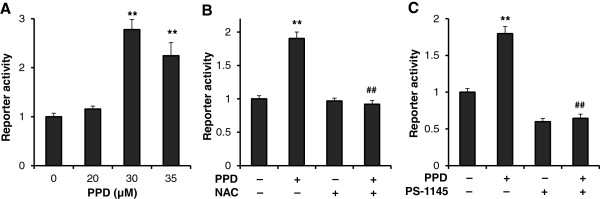
**PPD activated the NF-κB pathway via ROS generation.** (**A**) 24 h after transient transfection of NF-κB reporter plasmids, HCT-116 cells were treated with different concentrations of PPD for 24 h before the luciferase reporter activity was determined. (**B**) HCT-116 cells were treated with 35 μM of PPD for 24 h in the presence or absence of NAC (10 mM). (**C**) HCT-116 cells were treated with 35 μM of PPD for 24 h in the presence or absence of PS-1145 (50 μM). Data are presented as the mean ± standard error. **P < 0.01 vs. control; ##*P* < 0.01 vs. PPD only.

Ginseng-induced ROS promotes cell survival in colorectal cancer cells via activation of the NF-κB pathway. To determine whether PPD-induced ROS also activates the NF-κB pathway, we measured NF-κB reporter activity after treating HCT-116 cells with PPD. At the same concentration that led to high levels of ROS (Figure 5), PPD significantly induced NF-κB reporter activity (Figure 6A).

Decreasing the level of ROS in the cell should decrease NF-κB reporter activity, assuming that increased levels of ROS are required to induce NF-κB signaling. Addition of the antioxidant NAC significantly blocked the PPD-mediated induction of NF-κB transcriptional activity (Figure 6B) and increased cell death in HCT-116 cells, suggesting that PPD-induced ROS contribute to the activation of the NF-κB pathway.

We expected that the activated NF-κB pathway would counteract PPD-induced cell death in colorectal cancer cells because a decrease in ROS decreases NF-κB transcriptional activity. To further test the relationship of PPD-induced NF-κB activation and cell death, we inhibited the NF-κB pathway by adding PS-1145, a specific inhibitor of the NF-κB pathway, to PPD-treated HCT-116 cells. PS-1145 significantly inhibited both basal and PPD-induced NF-κB transcriptional activity (Figure 6C), and increased PPD-induced cell death in HCT-116 cells. PPD-induced ROS contributed to the survival of colorectal cancer cells via activation of the NF-κB pathway. Therefore, PPD-induced colorectal cancer cell death is enhanced by antioxidants or an NF-κB inhibitor.

## Discussion

After several years of cultivation in China or Korea, the root of Asian ginseng is harvested and steamed at standard boiling temperature; the steamed root is called red ginseng. It is believed that the steaming changes the ginsenoside composition profile, improving pharmacological activities. Our group used controlled variable steaming parameters to treat American ginseng and notoginseng to obtain desired ginsenoside profiles for increasing cancer chemopreventive effects [14,15].

American ginseng is a commonly used dietary supplement in United States. The major ginsenosides in American ginseng belong to the protopanaxadiol group and include Rb1, Rc and Rd [23]. Ginsenoside Rb1 is the most abundant ginsenoside in American ginseng, accounting for over 50% of total ginsenosides in many samples. Figure 1 elucidates the ginsenoside transformation by steaming treatment and intestinal microbiota. During steaming treatment, the protopanaxadiol group of ginsenosides are mainly transformed via three pathways: 1) to Rg5 and then to Rh4; 2) to Rk1 and then to Rk3; and 3) a major route, to Rg3, Rh2, and then to PPD [15,24]. Interestingly, after oral ginseng administration, gut microbiota, which normally exist in the gastrointestinal tract, can convert Rb1 and other protopanaxadiol group ginsenosides into their metabolite, ginsenoside compound K (CK) [25,26]. Recently, our study data showed that a relatively high amount of CK could be transformed to PPD by gut microbiota (unpublished data). Thus, the evaluation of cancer chemopreventive effects of PPD and its related mechanism of action is particularly pertinent to ginseng anticancer investigations.

Two representative human cancer cell lines, HCT-116 and SW-480, which differ in the expression of the p53 tumor suppressor gene, were used in our cell death observation. HCT-116 is p53 wild-type, while SW-480 has mutated p53. Tumor suppressor gene p53 is thought to be important in the cellular response to chemotherapeutic agent-induced damage. Mutation in p53 has been shown to correlate with an increased resistance to chemotherapy in cancer cells [27]. Our results showed that PPD significantly induced cell death in these two cell lines in a concentration-dependent manner, and HCT-116 was more sensitive even at lower concentrations. After PPD treatment, accumulation of cytoplasmic vacuoles was observed in the cancer cells, suggesting that paraptosis, another type of programmed cell death [19], was associated with PPD’s effect. Paraptosis is further supported by a mitochondrial swelling assay and by the observation that PPD used in combination with cycloheximides, a protein synthesis inhibitor, blocked PPD-induced vacuole formation.

5-Fluorouracil (5-FU) is a chemotherapeutic agent widely used for cancers of different organs including colorectum [28]. The clinical response rate of 5-FU in colorectal cancer treatment was 20-30%, and in combination with irinotecan or oxaliplatin, up to 50% [29,30]. Consistent with others [31], we observed that the cell death induction of 5-FU is not very impressive, with apoptosis as a main cellular response to treatment with 5-FU. At comparable concentrations, our data showed that PPD more significantly induced cell death. It is crucial for future work to identify combinatory therapies of natural product-derived compounds to increase the effectiveness of 5-FU on human colorectal cancer.

Our data showed that in both tested colon cancer cell lines, PPD significantly induced ROS production. Further, PPD activated the NF-κB pathway, which is linked to ROS generation. When the antioxidant NAC was used, NF-κB reporter activity levels were significantly reduced. This observation suggested that co-administration of PPD and a botanical antioxidant may achieve better cancer chemopreventive effects. Panaxadiol (PD) is a purified sapogenin of ginseng saponins, which has exhibited significant anticancer activity [11]. Structurally, PD is very similar to PPD, with basically only side chain cyclization [23]. Epigallocatechin gallate (EGCG), a major catechin in green tea, is a strong botanical antioxidant. Recently, we reported synergistic anticancer effects of PD and EGCG [32]. Based on the similar chemical structure of PD and PPD, it would be expected that co-administration of PPD and EGCG could also initiate increased cancer chemoprevention activities.

## Conclusions

Results from the present study suggest that paraptosis and NF-κB activation are associated with PPD-induced cancer chemoprevention. The activation of PPD’s NF-κB pathway is linked to its increased ROS level.

## Competing interests

The authors declare that they have no competing interests.

## Authors’ contributions

CZW, TCH, WD and CSY designed the study, set up the experiments, participated in data collection, analyzed and interpreted the results, and drafted the manuscript. BL, XDW, ZZ and CY carried out experiments and participated in data interpretation. TDC edited the manuscript. All authors read and approved the final manuscript.

## Pre-publication history

The pre-publication history for this paper can be accessed here:

http://www.biomedcentral.com/1472-6882/13/2/prepub
